# QSM-detected iron accumulation in the cerebellar gray matter is selectively associated with executive dysfunction in non-demented ALS patients

**DOI:** 10.3389/fneur.2024.1426841

**Published:** 2024-09-19

**Authors:** Edoardo Nicolò Aiello, Valeria Elisa Contarino, Giorgio Conte, Federica Solca, Beatrice Curti, Alessio Maranzano, Silvia Torre, Silvia Casale, Alberto Doretti, Eleonora Colombo, Federico Verde, Vincenzo Silani, Chunlei Liu, Claudia Cinnante, Fabio Maria Triulzi, Claudia Morelli, Barbara Poletti, Nicola Ticozzi

**Affiliations:** ^1^Department of Neurology and Laboratory of Neuroscience, IRCCS Istituto Auxologico Italiano, Milano, Italy; ^2^Neuroradiology Unit, Fondazione IRCCS Ca’ Granda Ospedale Maggiore Policlinico, Milano, Italy; ^3^Department of Pathophysiology and Transplantation, "Dino Ferrari" Center, Università degli Studi di Milano, Milano, Italy; ^4^Department of Electrical Engineering and Computer Sciences, University of California, Berkeley, Berkeley, CA, United States; ^5^Department of Diagnostic Imaging, IRCCS Istituto Auxologico Italiano, Milano, Italy; ^6^Department of Oncology and Hemato-Oncology, Università degli Studi di Milano, Milano, Italy

**Keywords:** quantitative susceptibility imaging, amyotrophic lateral sclerosis, cerebellum, frontotemporal degeneration, executive functioning

## Abstract

**Background:**

This study aimed to assess whether quantitative susceptibility imaging (QSM)-based measures of iron accumulation in the cerebellum predict cognitive and behavioral features in non-demented amyotrophic lateral sclerosis (ALS) patients.

**Methods:**

A total of ALS patients underwent 3-T MRI and a clinical assessment using the ALS Functional Rating Scale-Revised (ALSFRS-R) and the Edinburgh Cognitive and Behavioural ALS Screen (ECAS). Regression models were applied to each subscale of the cognitive section of the ECAS and the ECAS-Carer Interview to examine the effect of QSM-based measures in white and gray matter (WM; GM) of the cerebellum, separately for right, left, and bilateral cerebellar regions of interest (ROIs). These effects were compared to those of cerebellar volumetrics in WM/GM, right and left hemispheres while controlling for demographics, disease status, and total intracranial volume.

**Results:**

Higher QSM measures of the cerebellar GM on the left, right, and bilateral sides significantly predicted (*p*s ≤ 0.003) a greater number of errors on the executive functioning (EF) subscale of the ECAS (ECAS-EF). Moreover, higher GM-related, QSM measures of the cerebellum were associated with an increased probability of a below-cut-off performance on the ECAS-EF (*p*s ≤ 0.024). No significant effects were observed for QSM measures of the cerebellar WM or for volumetric measures on the ECAS-EF. Other ECAS measures showed no significant effects. Bilateral QSM measures of the cerebellar GM also selectively predicted performance on backward digit span and social cognition tasks.

**Discussion:**

Iron accumulation within the cerebellar GM, particularly in the cerebellar cortices, may be associated with executive functioning deficits in non-demented ALS patients. Therefore, QSM-based measures could be useful for identifying the neural correlates of extra-motor cognitive deficits in ALS patients.

## 1 Background

Among the non-pyramidal abnormalities observed in amyotrophic lateral sclerosis (ALS) (1), cerebellar involvement has recently been proposed as a key contributor to the phenotypic heterogeneity of the disease, possibly due to the cerebellum’s extensive connections with several encephalic and spinal sites (2). It has been suggested that an interplay between primary degenerative processes affecting the cerebellum and its compensatory role for brain/spinal cord dysfunctions may be the pathophysiological mechanism through which cerebellar involvement contributes to the clinical manifestations of ALS (2).

While the association between cerebellar involvement and motor manifestations in ALS has been sufficiently explored (3, 4), less is known on its role towards non-motor, neuropsychological features (2). Despite being poorly understood how cerebellar abnormalities contribute to cognition and behaviour in brain disorders (5), a number of reports would suggest they do account, at least to an extent, for both cognitive (6–8) and behavioural dysfunctions (6, 9, 10) in ALS – likely due to an altered connectivity between cerebellar nuclei and extra-motor networks (11).

While currently available studies on the topic mostly addressed morphological/functional neuroimaging techniques (2), none of them employed quantitative susceptibility mapping (QSM) algorithms (12) – although such an approach has proved promising in delivering a biomarker of subtle brain abnormalities underlying motor signs/symptoms in ALS (13–16). QSM algorithms indeed allow to derive an estimate of iron accumulation in brain tissues based on magnetic resonance imaging (MRI) scans, this being a feature common to several neurodegenerative diseases (17). Relatedly, it has been shown that QSM measures do relate to neuropsychological measures in such conditions (17), having thus been suggested as a promising marker for cognitive decline in several brain disorders (18).

A growing body of evidence has indicated that iron overload may play a causal role in neuronal death, leading to neurodegeneration (19, 20). Thus, QSM analyses might serve as a valuable tool for detecting subtle alterations in brain tissue iron content before regional brain atrophy occurs as an epiphenomenon of neurodegeneration (21), including in conditions such as ALS (16). This is particularly relevant for cerebellar involvement, in ALS, which is often subtle and may go undetected by standard volumetric measurements (8). Within such a framework, it would not be unreasonable to hypothesize that QSM algorithms might enhance the study of the relationships between cerebellar involvement and neuropsychological features in this population.

Given the above premises, the present study aimed to assess whether cerebellar QSM measures, compared to standard volumetric MRI measures, can predict cognitive and behavioral features in non-demented ALS patients.

## 2 Methods

### 2.1 Participants

Data from 61 consecutive ALS patients diagnosed according to the El Escorial criteria (22) and referred to IRCCS Istituto Auxologico Italiano between 2016 and 2021 were retrospectively retrieved. Patients with available MRI and neuropsychological data, specifically Edinburgh Cognitive and Behavioural ALS Screen (ECAS) scores (23), were selected for inclusion. No patient met the criteria for any frontotemporal degeneration phenotype (24, 25). The exclusion criteria included (1) the presence of other neurological/psychiatric disorders, (2) severe or uncompensated general medical conditions (i.e., internal/metabolic diseases or organ/system failures), or (3) uncorrected hearing/vision deficits. This study was approved by the Ethical Committee of IRCCS Istituto Auxologico Italiano (I.D.: 23C722_2017). Informed consent was obtained from all participants, and data were treated in accordance with current regulations.

### 2.2 Materials

#### 2.2.1 Clinical assessment

The ALS Functional Rating Scale-Revised was employed to assess motor-functional outcomes (26). Cognition and behavior assessments were conducted using the cognitive section of the ECAS (23) and the ECAS-Carer Interview (ECAS-CI) (27), respectively. The cognitive section of the ECAS is a performance-based screening tool ranging from 0 to 136, comprising five subscales that assess both ALS-specific cognitive function, including language (ECAS-L; *range* = 0–28), verbal fluency (ECAS-F; *range* = 0–24), and executive functioning (ECAS-EF; *range* = 0–28), as well as ALS-nonspecific cognitive functions, such as memory (ECAS-M; *range* = 0–24) and visuospatial abilities (ECAS-VS; *range* = 0–12) (23). The ECAS-CI is a 13-item, Likert-scaled, caregiver-report questionnaire that covers the full range of frontotemporal and dysexecutive behavioral features typical of ALS; higher scores indicate a greater degree of behavioral involvement (27).

#### 2.2.2 MRI acquisition

The patients underwent brain MRI scans using a 3-T SIGNA (General Electric, GE Healthcare Medical Systems, Chicago, Illinois, US) at the Istituto Auxologico Italiano, IRCCS, Milano, Italy. The MR protocol included the following sequences: a whole-brain sagittal three-dimensional FSPGR BRAVO T1-weighted sequence (TR = 8.7 ms, TE = 3.2 ms, inversion time = 450 ms, voxel size = 1 × 1 × 1 mm^3^, flip angle = 12°, acquisition matrix 256 × 256); a sagittal 3D fluid-attenuated inversion recovery (FLAIR) sequence (TR = 6,000 ms, TE = 108 ms, inversion time = 1,824 ms, voxel size = 1 × 1 × 1.4 mm^3^, flip angle = 90°, acquisition matrix 224 × 224); an axial T2-weighted fast spin-echo (FSE) sequence (TR = 3,000 ms, TE = 82 ms, pixel size = 0.234 × 0.234 mm^2^, slice thickness = 2 mm, slice gap = 0.2 mm, flip angle = 111°, acquisition matrix 320 × 320); and three-dimensional spoiled gradient-echo multi-echo (GRE) pulse sequences (TR = 39 ms, TE = 24 ms, delta TE = 3.3 ms, number of echoes = 7, voxel size = 0.468 × 0.468 × 1.4 mm^3^, flip angle = 20°, acquisition matrix 416 × 320).

Images were assessed by an expert neuroradiologist and were found to be free of motion and severe artifacts on T1-weighted or GRE images.

#### 2.2.3 MRI processing

**Figure 1:**
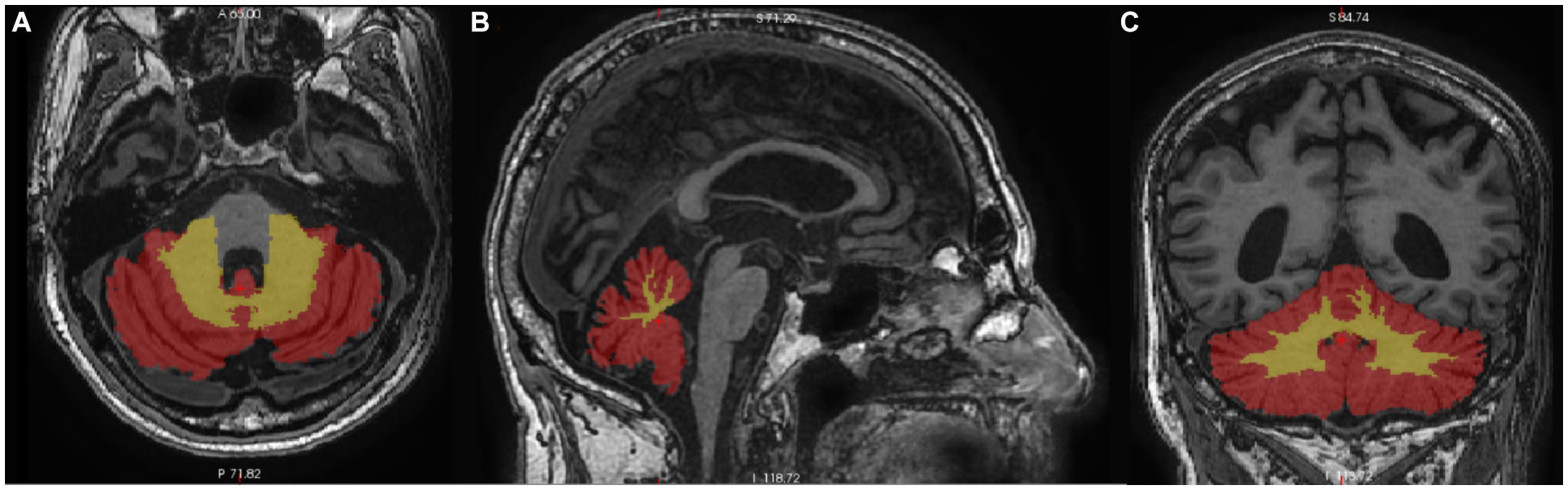
Axial **(A)**, sagittal **(B)**, and coronal **(C)** view of the cerebellum segmented on T1-weighted images. Cerebellar gray matter is colored red, and cerebellar white matter is yellow.

FreeSurfer was used to automatically segment T1-weighted images (28). The *Left-Cerebellum-Cortex* (coded “8”) and *Right-Cerebellum-Cortex* (coded “47”) regions were merged to create the *CerebellumGM* Region of Interest (ROI). Similarly, the *Left-Cerebellum-White-Matter* (coded “7”) and *Right-Cerebellum-White-Matter* (coded “46”) regions were merged to create the *CerebellumWM* ROI, as shown in Figure 1. The volumes of the *CerebellumGM* and *CerebellumWM* ROIs were measured in cubic millimeters (mm^3^).

Whole-brain QSM (quantitative susceptibility mapping) was calculated using STI Suite, a MATLAB toolbox specifically developed at UC Berkeley for MRI phase and magnetic susceptibility mapping (29). First, a single-subject brain mask was generated from the echoes-averaged magnitude image using the FSL Brain Extraction Tool (30). Then, the VSHARP algorithm was applied to remove the background field from phase images using the brain mask, followed by the application of the Streaking Artifacts Reduction (STAR) algorithm (31) to the VSHARP filtered phase images, resulting in subject-level susceptibility maps (32).

**Figure 2:**
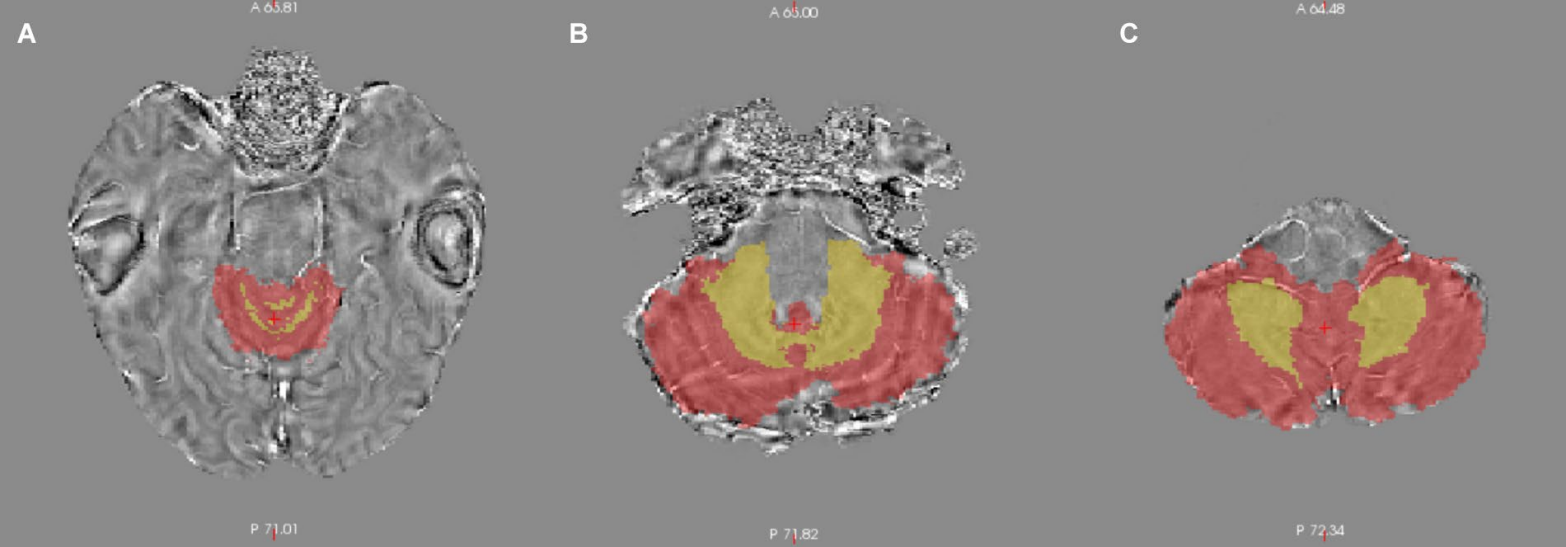
Superior **(A)**, middle **(B)**, and inferior **(C)** axial slices of the segmented cerebellum overlapped on the quantitative susceptibility map.

The SPM12 MATLAB Toolbox was used to coregister the QSM map to the T1-weighted image. For each patient, a transformation matrix was calculated by aligning the magnitude image to the T1-weighted image, which was then applied to the QSM image. The *CerebellumGM* and *CerebellumWM* ROIs overlaid on the coregistered QSM map (Figure 2). MATLAB (The MathWorks, Natick, MA) R2018 was used to extract the mean of the QSM values for the CerebellumGM and CerebellumWM ROIs in the left and right hemispheres separately, as well as for the total bilateral ROIs.

The neuroimaging pipeline was adapted from a previous QSM study focused on the precentral cortex (13).

### 2.3 Statistics

Both cognitive and behavioral measures did not meet the linear model assumptions (i.e., normality and homoscedasticity), as indexed by excessive skewness and values (i.e., ≥ |1| and |3|, respectively) (33), visual abnormalities in Q-Q plots and histograms, and significant Shapiro–Wilk’s tests (*p*s < 0.001). Specifically, ECAS-L, -F, -EF, -M, and-VS scores were left-skewed (i.e., showed ceiling effects), whereas ECAS-CI scores were right-skewed (i.e., showed floor effects). Additionally, both cognitive and behavioral measures showed overdispersion, meaning that they were highly variable between individuals. To address these issues, predictions of interests were tested using negative binomial (NB) regressions, which are suitable for modeling left-skewed and overdispersed count-like data (34) and have been shown to effectively model ALS patients’ cognitive/behavioral data (27, 35, 36).

For the ECAS-L/-F/-EF/-M/-VS subscales, NB regression was applied by focusing on the number of errors on each subscale as the outcome (37). This approach reversed the skewness of the empirical distribution without altering the original operationalization of the data (i.e., accuracy) (38). Moreover, the NB model was chosen over linear models with normalized and variance-stabilized transformed outcomes, as those transformations would have changed the original measurement units of the outcomes. In contrast, ECAS-CI scores already followed an NB distribution and, thus, did not require any transformation.

NB regressions were conducted for each subscale of the cognitive section of the ECAS and the ECAS-CI to test the effect of QSM-based white and gray matter (WM; GM) cerebellar measures, separately for right, left, and bilateral cerebellar ROIs (resulting in three models for each outcome). These were compared to WM/GM measures and right and left cerebellar volumetrics. Within these models, demographic variables (age, education, and sex), disease duration (in months), ALSFRS-R scores, presence of *C9orf72* hexanucleotide repeat expansion, and total intracranial volume were included as covariates.

For those NB regressions that identified significant QSM predictors, a logistic regression (LR) was subsequently performed to assess above versus. Below-cut-off performance on the relevant ECAS measure of interest (23). Poletti et al.’s (23) cut-offs were derived using a 2 SD-based approach and are stratified by age and education level (i.e., ≤60 and > 60 years of age and < 14 and ≥ 14 years of education, respectively). Accordingly, given that ECAS cut-offs are already adjusted for age and education, these covariates were not included in the LR models. Collinearity within the LR models was diagnosed if the variance inflation factor (VFI) exceeded 10 or if the tolerance index was less than 0.1 (39).

Missing data were excluded on a pairwise basis. The significance level was set at α = 0.05, with Bonferroni-corrected applied to target predictors whenever adequate. Analyses were conducted using jamovi 2.5.^1^

## 3 Results

Table 1 summarizes the patients’ background, clinical, volumetric, and QSM-based measures.

**Table 1:** Patients’ background, clinical and neuroradiological features.

*N*	61
Age (years)	63.9 ± 10.5 (41–84)
Sex (male/female)	34/27
Education (years)	11.1 ± 4.6 (5–23)
Disease duration (months)	20.4 ± 34.1 (2–264)
ALSFRS-R	38.5 ± 6.2 (23–48)
ΔFS	0.9 ± 1 (0–5.2)
NIV (%)	5.1%
PEG (%)	0%
Genetics (*N*)	
*C9orf72/TARDBP*	5/3
ECAS	
Total	99.9 ± 17.6 (47–127)
Impaired (%)	31.1%
Language	23.5 ± 4 (12–28)
Impaired (%)	24.6%
Fluency	16.3 ± 5.8 (0–24)
Impaired (%)	16.4%
Executive	34.6 ± 7.6 (13–48)
Impaired (%)	21.3%
Memory	14.3 ± 4.8. (2–21)
Impaired (%)	24.6%
Visuospatial	11.3 ± 1.2 (6–12)
Impaired (%)	9.8%
Carer Interview	0.7 ± 0.8 (0–3)
Abnormal (%)	2%
Total ICV (mm^3^)	1508913.1 ± 159358.8 (1251380.9–1879999.7)
Cerebellar volumes (mm^3^)	
Gray matter	
Right hemisphere	53,480 ± 6,085 (41364.2–64792.5)
Left hemisphere	53416.2 ± 5693.6 (41475.3–65381.8)
White matter	
Right hemisphere	13124.6 ± 2647.4 (8866.2–24547.5)
Left hemisphere	13431.5 ± 2005.3 (9446.2–18434.3)
Cerebellar QSM measures	
Gray matter	
Right hemisphere	−1.4 ± 1 (−3.6–1.1)
Left hemisphere	−1.1 ± 0.9 (−3.1–1.3)
Bilateral	−1.2 ± 0.8 (−2.9–1.1)
White matter	
Right hemisphere	−0.5 ± 1.3 (−3.6–2.2)
Left hemisphere	−0.2 ± 1.3 (−3.4–3.2)
Bilateral	−0.3 ± 1.1 (−3.2–2.3)

ALS, amyotrophic laterals sclerosis; ECAS, Edinburgh Cognitive and Behavioural ALS Screen; ALSFRS-R, Amyotrophic Lateral Sclerosis Functional Rating Scale-Revised; ΔFS, progression rate, computed as: [(48-ALSFRS-R)/disease duration in months]; NIV, non-invasive ventilation; PEG, percutaneous endoscopic gastrostomy; QSM, quantitative susceptibility mapping.

**Figure 3:**
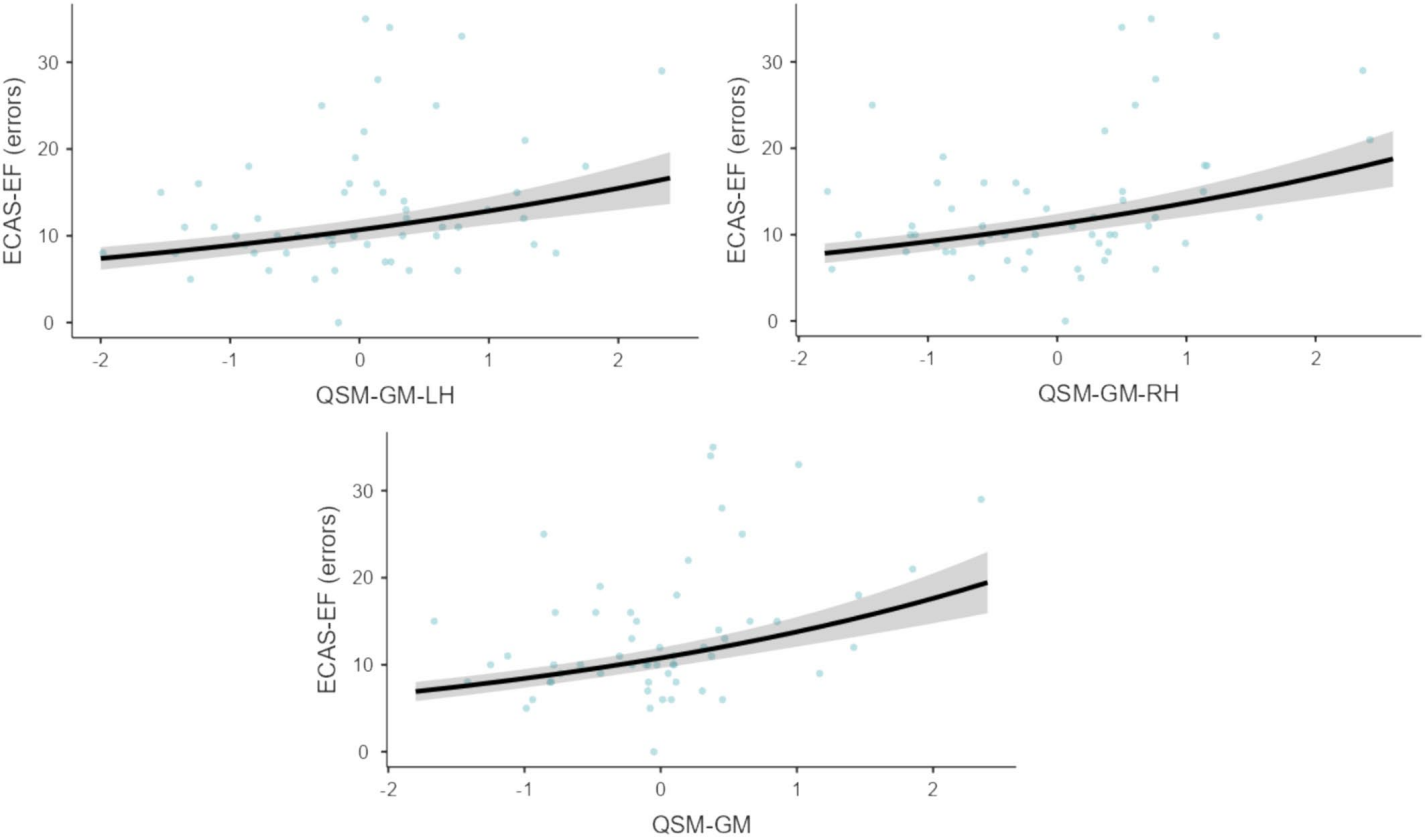
Predictions of left- (upper-left panel) and right-sided (upper-right panel), as well as bilateral (lower panel) QSM-based measures of the cerebellum towards the ECAS-EF. ECAS, Edinburgh Cognitive and Behavioural ALS Screen; EF, Executive functioning; QSM, quantitative susceptibility mapping; WM, white matter; GM, gray matter; LH, left hemisphere; RH, right hemisphere. Gray areas represent the SE of the estimate of the Negative Binomial regression (left-sided: *z* = 2.97; *p* = 0.003; right-sided: *z* = 3.75; *p* < 0.001; bilateral: *z* = 3.95; *p* < 0.001). The measurement unit on the *x*-axis is ppb; these predictors have been mean-centered for this graphical representation.

As shown in Table 2, no significant effects were observed for either QSM-based or volumetric cerebellar measures on ECAS-L, ECAS-F, ECAS-M, ECAS-VS, or ECAS-CI scores. However, higher QSM measures of the cerebellar GM, on both the left and right sides, as well as the total mean QSM measures, were found to selectively predict a higher number of errors on the ECAS-EF subscale (Figure 3). In contrast, neither QSM-based measures of the cerebellar WM nor volumetric cerebellar measures significantly predicted ECAS-EF errors (Table 2). Supplementary Tables 1–6 provide the full results of the NB models that address bilateral QSM-based and volumetric measures.

**Table 2:** The negative binomial regressions yielded the effects of QSM-based and volumetric measures on the number of errors on the cognitive subscales of the ECAS and the ECAS-CI.

Model			ECAS-L	ECAS-F	ECAS-EF	ECAS-M	ECAS-*VS*	ECAS-CI
LH^1^	QSM	WM	0.320	0.144	0.938	0.902	0.318	0.792
		GM	0.925	0.165	**0.003**	0.294	0.369	0.847
	Volume	WM	0.833	0.141	0.988	0.512	0.138	0.968
		GM	0.441	0.573	0.904	0.315	0.197	0.757
RH^1^	QSM	WM	0.826	0.939	0.349	0.175	0.832	0.625
		GM	0.273	0.792	**<0.001**	0.995	0.090	0.376
	Volume	WM	0.334	0.820	0.753	0.488	0.035	0.937
		GM	0.824	0.803	0.735	0.225	0.083	0.788
Bilateral	QSM	WM	0.425	0.397	0.796	0.328	0.618	0.840
		GM	0.645	0.416	**<0.001**	0.789	0.241	0.475
	Volume-LH	WM	0.797	0.169	0.941	0.785	0.457	0.773
		GM	0.098	0.127	0.563	0.880	0.085	0.351
	Volume-RH	WM	0.659	0.530	0.870	0.874	0.086	0.818
		GM	0.146	0.129	0.611	0.459	0.377	0.336

^1^αadjusted = 0.013. ^2^αadjusted = 0.008. Each model comprised sex, age, education, presence of *C9orf72* hexanucleotide repeat expansion, disease duration (in months), ALS Functional Rating Scale-Revised scores, and total intracranial volume as covariates; *p*-values refer to the *χ*^2^-statistics for the main effect of each term (significant ones are in bold). ECAS, Edinburgh Cognitive and Behavioural ALS Screen; L, Language; F, Fluency; EF, Executive functioning; M, Memory; VS, Visuo-spatial; CI, Carer Interview; QSM, quantitative susceptibility mapping; WM, white matter; GM, gray matter; LH, left hemisphere; RH, right hemisphere.

LRs conducted on above-vs. below-cut-off ECAS-EF performance confirmed that higher GM-related, QSM-based measures of the cerebellum were associated with a higher likelihood of a below-cut-off ECAS-EF performance (right-sided: *b* = 3.72, *z* = 2.41, *p* = 0.016; left-sided: *b* = 1.85, *z* = 2.36, *p* = 0.018; total: *b* = 4.36, *z* = 2.06, *p* = 0.034). In contrast, neither QSM-based measures of the cerebellar WM nor volumetric measures have any significant effects (*p*s ≥ 0.538 and *p*s ≥ 0.197, respectively). No collinearity issues were detected in the models addressing right-and left-sided measures; however, collinearity was observed between right-and left-sided GM and WM volumetric measures (VFI ≥ 14.8; tolerance index≤0.07) in the model addressing total mean QSM measures. Supplementary Table 7 shows the full results of the LR model addressing bilateral QSM-based and volumetric measures.

Finally, an explanatory, data-driven set of regression models was conducted to test which specific ECAS-EF tasks (i.e., backward digit span, alternation, sentence completion, and social cognition) could be predicted by QSM measures. To this end, each ECAS-EF task was regressed using the set of predictors and covariates used in the abovementioned NB models addressing bilateral cerebellar ROIs. However, models including only right or left cerebellar measures were not conducted, as no laterality effects were used in the analyses addressing the ECAS-EF as a whole. Specifically, linear regressions were used for backward digit span and sentence completion scores due to their normal distribution, while NB regressions were used for alternation and social cognition tasks, with the number of errors as the outcome variable.

Table 3 presents the results of these off-label analyses. Overall, only *backward digit span* (*b* = −0.74; *z* = −2.69; *p* = 0.010) and *social cognition* performances (*b* = 0.90, *z* = 2.89, *p* = 0.004) were significantly predicted by QSM measures of the cerebellar GM. No significant effects were found for QSM measures of the cerebellar WM or volumetric measures. Supplementary Tables 8–11 provide the full results of the NB models addressing bilateral QSM-based and volumetric measures.

**Table 3:** The effects of QSM-based and volumetric measures on each task of the ECAS-EF were yielded by linear and negative binomial regressions.

Model			Backward digit span*	Alternation^§^	Sentence completion^*^	Social cognition^§^
Bilateral^1^	QSM	WM	0.699	0.807	0.218	0.453
		GM	**0.007**	0.338	0.375	**0.002**
	Volume-LH	WM	0.034	0.561	0.782	0.251
		GM	0.394	0.349	0.988	0.934
	Volume-RH	WM	0.690	0.180	0.595	0.653
		GM	0.362	0.329	0.900	0.624

Each model comprised sex, age, education, presence of *C9orf72* hexanucleotide repeat expansion, disease duration (in months), ALS Functional Rating Scale-Revised scores, and total intracranial volume as covariates; *p*-values refer to the *χ*^2^-statistics for the main effect of each term (significant ones are in bold). ECAS, Edinburgh Cognitive and Behavioural ALS Screen; EF, executive functioning; QSM, quantitative susceptibility mapping; WM, white matter; GM, gray matter; LH, left hemisphere; RH, right hemisphere.

1 α_adjusted_ = 0.008.

* Linear regression model.

§ Negative binomial model.

## 4 Discussion

The present study provides, for the first time, promising evidence of the predictive capability of QSM-detected iron accumulation within the cerebellar GM in relation to executive functioning (EF) deficits in non-demented ALS patients.

Notably, this predictive capability (1) was not observed in QSM-based measures and (2) did not apply to common volumetrics of the cerebellum. Such selectivity of QSM-based measures, as opposed to macroscopic volumetric ones, aligns with the idea that tissue alteration, such as iron overload, may precede brain atrophy (19, 20).

However, it is somewhat surprising that these findings were significant for cerebellar GM regions of interest (ROIs) but not for WM ROIs, which, in this study using Freesurfer segmentation, included the cerebellar nuclei. This is unexpected, given that both cerebellar nuclei and cortical-cerebellar connections have frequently been shown to underlie the clinical phenotype of ALS (2), rather than the cerebellar cortex. This raises the possibility that, in non-demented ALS patients, the cerebellar cortices also play a role in their cognitive profiles. Given the novelty of these results, further research is needed to determine whether the cerebellar cortex is selectively involved in characterizing the cognitive features of non-demented ALS patients.

In the present study, QSM-based measures of cerebellar GM selectively predicted EF measures but did not predict other cognitive functions or behaviors. This finding aligns with the current knowledge of the cerebellum’s contributions to cognition in both healthy and brain-damaged individuals, where the cerebellum is known to be substantially involved in EF (40, 41). However, previous studies on neurodegenerative conditions other than ALS have not detected a link between cognitive features and QSM-detected iron accumulation in the cerebellum (17, 18). This unprecedented finding highlights the need for further research into the role of cerebellar iron accumulation in cognitive impairment across neurodegenerative disorders, including ALS.

In this respect, it is also worth mentioning that a set of off-label analyses revealed that QSM-based measures of the cerebellar GM selectively predicted performance on *backward digit span tasks*, which are related to phonological working memory and *social cognition* tasks. The first finding aligns with previous neuroimaging studies in both healthy (42, 43) and brain-damaged populations (44, 45), which have linked phonological working memory abilities to the integrity of cerebellar structures. Similarly, the current link between social-cognitive abilities and iron accumulation in the cerebellar GM is consistent with the widely accepted idea that the cerebellum supports such a set of high-order skills by storing “social scripts,” which help individuals predict social interactions (46).

The present study is not free of limitations. First, the current investigation merely focuses on the cerebellum, which limits our ability to determine whether iron accumulation in other structures, either supratentorial or supratentorial, might also be associated with EF in the present cohort. Future research should explore whether the association between iron accumulation and FTD-*spectrum* cognitive deficits in ALS patients is specific to the cerebellum or extends to other brain regions.

Second, the lack of a control group prevented us from assessing whether QSM-based measures are similarly associated with executive functioning in healthy individuals. Third, the sample was relatively limited in size, possibly undermining the generalizability of the present findings. Fourth, the patients included were not stratified according to Strong et al.’s (47) criteria, and there were no cases of ALS with comorbid frontotemporal degeneration. Future studies should aim to replicate these analyses with a larger, more representative, and well-characterized sample of ALS patients.

Fifth, the cerebellum was segmented based on broad dichotomies (i.e., GM vs. WM and left vs. right hemispheric lateralization). Cerebellar nuclei were included within the WM ROIs and were not individually analyzed, although, as previously stated, cerebellar nuclei have been frequently identified as relevant to the clinical characterization of ALS (2). Therefore, further studies are needed to focus on more detailed cerebellar ROIs. In this respect, it should also be noted that, instead of using FreeSurfer, other cerebellum-specific segmentation techniques, such as SUIT, could have been employed (48). While the current choice of FreeSurfer was motivated by its widespread use and acceptability among local collaborating Italian institutions, future studies should consider using more specialized segmentation approaches to replicate or challenge these findings. Finally, a statistical limitation must be highlighted: collinearity was identified among cerebellar volumetrics within the LR model addressing bilateral QSM-based measures. Although this collinearity was expected, it may have introduced some bias into the results of the abovementioned model.

In conclusion, iron accumulation within the cerebellar GM, i.e., in the cerebellar cortices, may be associated with EF deficits in non-demented ALS patients, thereby suggesting that cerebellar in this population might contribute, at least in part, to FTD-*spectrum* phenotypic features in this population. Additionally, this report is the first to demonstrate the potential usefulness of QSM-based measures in uncovering the neural correlates of extra-motor cognitive deficits in ALS.

## Data Availability

Datasets associated with the present study have been stored in an online repository (https://doi.org/10.5281/zenodo.13619839). These datasets cannot be made publicly available on ethical and legal grounds. Requests to access the datasets should be directed to the corresponding authors who will forward a request for a data transfer agreement to the relevant Ethical Committee.

## Footnotes

^1^ https://www.jamovi.org/
