# Supplementary material for: QSM-detected iron accumulation in the cerebellar gray matter is selectively associated with executive dysfunction in non-demented ALS patients
Source: Front Neurol. 2024 Sep 19;15:1426841. doi: 10.3389/fneur.2024.1426841 (PMC11448125; doi:10.3389/fneur.2024.1426841)
Supplement: Supplementary file 1 [file Table_1.DOCX]

| **Supplementary Table 1.** Results of the NB model addressing bilateral volumetric and QSM measures on the number of errors on the ECAS-Language. | | | | | | | | | | | | | | | | | |
| --- | --- | --- | --- | --- | --- | --- | --- | --- | --- | --- | --- | --- | --- | --- | --- | --- | --- |
|  | | | | | | | | | | **95% CI** | | | |  | | | |
| **Predictor** | | **Slope** | | ***b*** | | ***SE*** | | **OR** | | **LL** | | **UL** | | ***z*** | | ***p*** | |
| Sex |  | Female *vs.* Male |  | -0.289 |  | 0.254 |  | 0.749 |  | 0.460 |  | 1.213 |  | -1.139 |  | 0.255 |  |
| C9orf72 |  | Yes *vs.* No |  | -0.213 |  | 0.492 |  | 0.808 |  | 0.301 |  | 2.088 |  | -0.433 |  | 0.665 |  |
| Age (years) |  |  |  | 0.004 |  | 0.012 |  | 1.004 |  | 0.980 |  | 1.030 |  | 0.362 |  | 0.717 |  |
| Education (years) |  |  |  | -0.116 |  | 0.030 |  | 0.891 |  | 0.841 |  | 0.941 |  | -3.926 |  | < .001 |  |
| DD (months) |  |  |  | -0.002 |  | 0.004 |  | 0.998 |  | 0.988 |  | 1.005 |  | -0.522 |  | 0.602 |  |
| ALSFRS-R |  |  |  | 0.030 |  | 0.019 |  | 1.030 |  | 0.993 |  | 1.070 |  | 1.583 |  | 0.113 |  |
| Total ICV |  |  |  | -0.000 |  | 0.000 |  | 1.000 |  | 1.000 |  | 1.000 |  | -1.854 |  | 0.064 |  |
| Volume-WM-LH |  |  |  | -0.000 |  | 0.000 |  | 1.000 |  | 1.000 |  | 1.000 |  | -0.267 |  | 0.790 |  |
| Volume-GM-LH |  |  |  | 0.000 |  | 0.000 |  | 1.000 |  | 1.000 |  | 1.000 |  | 1.672 |  | 0.094 |  |
| Volume-WM-RH |  |  |  | 0.000 |  | 0.000 |  | 1.000 |  | 1.000 |  | 1.000 |  | 0.453 |  | 0.651 |  |
| Volume-GM-RH |  |  |  | -0.000 |  | 0.000 |  | 1.000 |  | 1.000 |  | 1.000 |  | -1.473 |  | 0.141 |  |
| QSM-WM |  |  |  | -0.088 |  | 0.108 |  | 0.915 |  | 0.736 |  | 1.138 |  | -0.819 |  | 0.413 |  |
| QSM-GM |  |  |  | 0.067 |  | 0.141 |  | 1.069 |  | 0.805 |  | 1.421 |  | 0.474 |  | 0.636 |  |
| **Notes.** NB=Negative Binomial; LL=lower limit; UL=upper limit; ECAS=Edinburgh Cognitive and Behavioural ALS Screen; DD=disease duration; ALSFRS-R=ALS Functional Rating Scale – Revised; ICV=intracranial volume; WM=white matter; LH=left hemisphere; GM=gray matter; RH=right hemisphere. | | | | | | | | | | | | | | | | | |

| **Supplementary Table 2.** Results of the NB model addressing bilateral volumetric and QSM measures on the number of errors on the ECAS-EF. | | | | | | | | | | | | | | | | | |
| --- | --- | --- | --- | --- | --- | --- | --- | --- | --- | --- | --- | --- | --- | --- | --- | --- | --- |
|  | | | | | | | | | | **95% CI** | | | |  | | | |
| **Predictor** | | **Slope** | | ***b*** | | ***SE*** | | **OR** | | **LL** | | **UL** | | ***z*** | | ***p*** | |
| Sex |  | Female *vs.* Male |  | -0.272 |  | 0.115 |  | 0.762 |  | 0.609 |  | 0.951 |  | -2.377 |  | 0.017 |  |
| C9orf72 |  | Yes *vs.* No |  | -0.223 |  | 0.231 |  | 0.800 |  | 0.504 |  | 1.246 |  | -0.967 |  | 0.334 |  |
| Age (years) |  |  |  | 0.008 |  | 0.006 |  | 1.008 |  | 0.997 |  | 1.019 |  | 1.401 |  | 0.161 |  |
| Education (years) |  |  |  | -0.049 |  | 0.013 |  | 0.953 |  | 0.928 |  | 0.977 |  | -3.715 |  | < .001 |  |
| DD (months) |  |  |  | -0.005 |  | 0.002 |  | 0.995 |  | 0.990 |  | 0.999 |  | -2.212 |  | 0.027 |  |
| ALSFRS-R |  |  |  | -0.007 |  | 0.008 |  | 0.994 |  | 0.978 |  | 1.010 |  | -0.799 |  | 0.424 |  |
| Total ICV |  |  |  | -0.000 |  | 0.000 |  | 1.000 |  | 1.000 |  | 1.000 |  | -3.187 |  | 0.001 |  |
| Volume-WM-LH |  |  |  | 0.000 |  | 0.000 |  | 1.000 |  | 1.000 |  | 1.000 |  | 0.075 |  | 0.941 |  |
| Volume-GM-LH |  |  |  | 0.000 |  | 0.000 |  | 1.000 |  | 1.000 |  | 1.000 |  | 0.578 |  | 0.563 |  |
| Volume-WM-RH |  |  |  | 0.000 |  | 0.000 |  | 1.000 |  | 1.000 |  | 1.000 |  | 0.165 |  | 0.869 |  |
| Volume-GM-RH |  |  |  | -0.000 |  | 0.000 |  | 1.000 |  | 1.000 |  | 1.000 |  | -0.511 |  | 0.610 |  |
| QSM-WM |  |  |  | -0.013 |  | 0.048 |  | 0.988 |  | 0.898 |  | 1.086 |  | -0.259 |  | 0.795 |  |
| QSM-GM |  |  |  | 0.246 |  | 0.062 |  | 1.279 |  | 1.133 |  | 1.443 |  | 3.948 |  | < .001 |  |
| **Notes.** NB=Negative Binomial; LL=lower limit; UL=upper limit; ECAS=Edinburgh Cognitive and Behavioural ALS Screen; EF=Executive Functioning; DD=disease duration; ALSFRS-R=ALS Functional Rating Scale – Revised; ICV=intracranial volume; WM=white matter; LH=left hemisphere; GM=gray matter; RH=right hemisphere. | | | | | | | | | | | | | | | | | |

| **Supplementary Table 3.** Results of the NB model addressing bilateral volumetric and QSM measures on the number of errors on the ECAS-Fluency | | | | | | | | | | | | | | | | | |
| --- | --- | --- | --- | --- | --- | --- | --- | --- | --- | --- | --- | --- | --- | --- | --- | --- | --- |
|  | | | | | | | | | | **95% CI** | | | |  | | | |
| **Predictor** | | **Slope** | | ***b*** | | ***SE*** | | **OR** | | **LL** | | **UL** | | ***z*** | | ***p*** | |
| Sex |  | Female *vs.* Male |  | -0.151 |  | 0.178 |  | 0.860 |  | 0.605 |  | 1.222 |  | -0.846 |  | 0.397 |  |
| C9orf72 |  | Yes *vs.* No |  | -0.109 |  | 0.373 |  | 0.897 |  | 0.423 |  | 1.828 |  | -0.292 |  | 0.770 |  |
| Age (years) |  |  |  | 0.007 |  | 0.009 |  | 1.007 |  | 0.990 |  | 1.025 |  | 0.829 |  | 0.407 |  |
| Education (years) |  |  |  | -0.099 |  | 0.020 |  | 0.905 |  | 0.870 |  | 0.941 |  | -4.938 |  | < .001 |  |
| DD (months) |  |  |  | 0.002 |  | 0.002 |  | 1.002 |  | 0.997 |  | 1.006 |  | 0.857 |  | 0.392 |  |
| ALSFRS-R |  |  |  | 0.009 |  | 0.013 |  | 1.009 |  | 0.983 |  | 1.035 |  | 0.675 |  | 0.500 |  |
| Total ICV |  |  |  | 0.000 |  | 0.000 |  | 1.000 |  | 1.000 |  | 1.000 |  | 0.639 |  | 0.523 |  |
| Volume-WM-LH |  |  |  | 0.000 |  | 0.000 |  | 1.000 |  | 1.000 |  | 1.000 |  | 1.353 |  | 0.176 |  |
| Volume-GM-LH |  |  |  | -0.000 |  | 0.000 |  | 1.000 |  | 1.000 |  | 1.000 |  | -1.505 |  | 0.132 |  |
| Volume-WM-RH |  |  |  | -0.000 |  | 0.000 |  | 1.000 |  | 1.000 |  | 1.000 |  | -0.611 |  | 0.541 |  |
| Volume-GM-RH |  |  |  | 0.000 |  | 0.000 |  | 1.000 |  | 1.000 |  | 1.000 |  | 1.524 |  | 0.128 |  |
| QSM-WM |  |  |  | 0.062 |  | 0.073 |  | 1.063 |  | 0.922 |  | 1.227 |  | 0.841 |  | 0.401 |  |
| QSM-GM |  |  |  | 0.081 |  | 0.100 |  | 1.084 |  | 0.892 |  | 1.317 |  | 0.811 |  | 0.417 |  |
| **Notes.** NB=Negative Binomial; LL=lower limit; UL=upper limit; ECAS=Edinburgh Cognitive and Behavioural ALS Screen; DD=disease duration; ALSFRS-R=ALS Functional Rating Scale – Revised; ICV=intracranial volume; WM=white matter; LH=left hemisphere; GM=gray matter; RH=right hemisphere. | | | | | | | | | | | | | | | | | |

| **Supplementary Table 4.** Results of the NB model addressing bilateral volumetric and QSM measures on the number of errors on the ECAS-Memory | | | | | | | | | | | | | | | | | |
| --- | --- | --- | --- | --- | --- | --- | --- | --- | --- | --- | --- | --- | --- | --- | --- | --- | --- |
|  | | | | | | | | | | **95% ci** | | | |  | | | |
| **Predictor** | | **Slope** | | ***b*** | | ***SE*** | | **OR** | | **LL** | | **UL** | | ***z*** | | ***p*** | |
| Sex |  | Female *vs.* Male |  | -0.113 |  | 0.143 |  | 0.893 |  | 0.674 |  | 1.183 |  | -0.791 |  | 0.429 |  |
| C9orf72 |  | Yes *vs.* No |  | 0.378 |  | 0.249 |  | 1.459 |  | 0.891 |  | 2.376 |  | 1.520 |  | 0.129 |  |
| Age (years) |  |  |  | 0.003 |  | 0.007 |  | 1.003 |  | 0.989 |  | 1.017 |  | 0.403 |  | 0.687 |  |
| Education (years) |  |  |  | -0.036 |  | 0.016 |  | 0.964 |  | 0.934 |  | 0.996 |  | -2.239 |  | 0.025 |  |
| DD (months) |  |  |  | -0.001 |  | 0.002 |  | 0.999 |  | 0.995 |  | 1.003 |  | -0.356 |  | 0.722 |  |
| ALSFRS-R |  |  |  | 0.004 |  | 0.010 |  | 1.004 |  | 0.984 |  | 1.026 |  | 0.432 |  | 0.666 |  |
| Total ICV |  |  |  | -0.000 |  | 0.000 |  | 1.000 |  | 1.000 |  | 1.000 |  | -1.481 |  | 0.139 |  |
| Volume-WM-LH |  |  |  | 0.000 |  | 0.000 |  | 1.000 |  | 1.000 |  | 1.000 |  | 0.274 |  | 0.784 |  |
| Volume-GM-LH |  |  |  | 0.000 |  | 0.000 |  | 1.000 |  | 1.000 |  | 1.000 |  | 0.151 |  | 0.880 |  |
| Volume-WM-RH |  |  |  | 0.000 |  | 0.000 |  | 1.000 |  | 1.000 |  | 1.000 |  | 0.162 |  | 0.872 |  |
| Volume-GM-RH |  |  |  | -0.000 |  | 0.000 |  | 1.000 |  | 1.000 |  | 1.000 |  | -0.744 |  | 0.457 |  |
| QSM-WM |  |  |  | 0.058 |  | 0.058 |  | 1.060 |  | 0.943 |  | 1.191 |  | 0.997 |  | 0.319 |  |
| QSM-GM |  |  |  | -0.022 |  | 0.081 |  | 0.979 |  | 0.834 |  | 1.146 |  | -0.268 |  | 0.789 |  |
| **Notes.** NB=Negative Binomial; LL=lower limit; UL=upper limit; ECAS=Edinburgh Cognitive and Behavioural ALS Screen; DD=disease duration; ALSFRS-R=ALS Functional Rating Scale – Revised; ICV=intracranial volume; WM=white matter; LH=left hemisphere; GM=gray matter; RH=right hemisphere. | | | | | | | | | | | | | | | | | |

| **Supplementary Table 5.** Results of the NB model addressing bilateral volumetric and QSM measures on the number of errors on the ECAS-Visuospatial. | | | | | | | | | | | | | | | | | |
| --- | --- | --- | --- | --- | --- | --- | --- | --- | --- | --- | --- | --- | --- | --- | --- | --- | --- |
|  | | | | | | | | | | **95% CI** | | | |  | | | |
| **Predictor** | | **Slope** | | ***b*** | | ***SE*** | | **OR** | | **LL** | | **UL** | | ***z*** | | ***p*** | |
| Sex |  | Female *vs.* Male |  | 0.386 |  | 0.533 |  | 1.471 |  | 0.520 |  | 4.297 |  | 0.724 |  | 0.469 |  |
| C9orf72 |  | Present *vs.* Absent |  | 1.065 |  | 0.907 |  | 2.901 |  | 0.463 |  | 17.538 |  | 1.175 |  | 0.240 |  |
| Age (years) |  |  |  | 0.013 |  | 0.025 |  | 1.013 |  | 0.965 |  | 1.065 |  | 0.520 |  | 0.603 |  |
| Education (years) |  |  |  | -0.166 |  | 0.068 |  | 0.847 |  | 0.733 |  | 0.960 |  | -2.442 |  | 0.015 |  |
| DD (months) |  |  |  | -0.008 |  | 0.012 |  | 0.993 |  | 0.960 |  | 1.009 |  | -0.643 |  | 0.520 |  |
| ALSFRS-R |  |  |  | 0.001 |  | 0.035 |  | 1.001 |  | 0.936 |  | 1.074 |  | 0.037 |  | 0.971 |  |
| Total ICV |  |  |  | -0.000 |  | 0.000 |  | 1.000 |  | 1.000 |  | 1.000 |  | -0.500 |  | 0.617 |  |
| Volume-WM-LH |  |  |  | 0.000 |  | 0.000 |  | 1.000 |  | 1.000 |  | 1.001 |  | 0.802 |  | 0.423 |  |
| Volume-GM-LH |  |  |  | 0.000 |  | 0.000 |  | 1.000 |  | 1.000 |  | 1.000 |  | 1.734 |  | 0.083 |  |
| Volume-WM-RH |  |  |  | -0.000 |  | 0.000 |  | 1.000 |  | 0.999 |  | 1.000 |  | -1.731 |  | 0.083 |  |
| Volume-GM-RH |  |  |  | -0.000 |  | 0.000 |  | 1.000 |  | 1.000 |  | 1.000 |  | -0.904 |  | 0.366 |  |
| QSM-WM |  |  |  | 0.105 |  | 0.216 |  | 1.111 |  | 0.733 |  | 1.685 |  | 0.485 |  | 0.627 |  |
| QSM-GM |  |  |  | 0.303 |  | 0.267 |  | 1.355 |  | 0.815 |  | 2.265 |  | 1.135 |  | 0.256 |  |
| **Notes.** NB=Negative Binomial; LL=lower limit; UL=upper limit; ECAS=Edinburgh Cognitive and Behavioural ALS Screen; DD=disease duration; ALSFRS-R=ALS Functional Rating Scale – Revised; ICV=intracranial volume; WM=white matter; LH=left hemisphere; GM=gray matter; RH=right hemisphere. | | | | | | | | | | | | | | | | | |

| **Supplementary Table 6.** Results of the NB model addressing bilateral volumetric and QSM measures on the ECAS-Carer Interview. | | | | | | | | | | | | | | | | | |
| --- | --- | --- | --- | --- | --- | --- | --- | --- | --- | --- | --- | --- | --- | --- | --- | --- | --- |
|  | | | | | | | | | | **95% CI** | | | |  | | | |
| **Predictor** | | **Slope** | | ***b*** | | ***SE*** | | **OR** | | **LL** | | **UL** | | ***z*** | | ***p*** | |
| Sex |  | Female *vs.* Male |  | -0.436 |  | 0.498 |  | 0.646 |  | 0.238 |  | 1.709 |  | -0.877 |  | 0.381 |  |
| C9orf72 |  | Yes *vs.* No |  | -1.512 |  | 1.161 |  | 0.220 |  | 0.011 |  | 1.543 |  | -1.303 |  | 0.193 |  |
| Age (years) |  |  |  | -0.014 |  | 0.022 |  | 0.986 |  | 0.944 |  | 1.030 |  | -0.628 |  | 0.530 |  |
| Education (years) |  |  |  | -0.012 |  | 0.055 |  | 0.988 |  | 0.881 |  | 1.097 |  | -0.221 |  | 0.825 |  |
| DD (months) |  |  |  | -0.015 |  | 0.014 |  | 0.985 |  | 0.948 |  | 1.001 |  | -1.079 |  | 0.280 |  |
| ALSFRS-R |  |  |  | -0.009 |  | 0.035 |  | 0.991 |  | 0.928 |  | 1.066 |  | -0.245 |  | 0.807 |  |
| Total ICV |  |  |  | -0.000 |  | 0.000 |  | 1.000 |  | 1.000 |  | 1.000 |  | -0.040 |  | 0.968 |  |
| Volume-WM-LH |  |  |  | -0.000 |  | 0.000 |  | 1.000 |  | 0.999 |  | 1.000 |  | -0.286 |  | 0.775 |  |
| Volume-GM-LH |  |  |  | 0.000 |  | 0.000 |  | 1.000 |  | 1.000 |  | 1.000 |  | 0.919 |  | 0.358 |  |
| Volume-WM-RH |  |  |  | 0.000 |  | 0.000 |  | 1.000 |  | 1.000 |  | 1.001 |  | 0.230 |  | 0.818 |  |
| Volume-GM-RH |  |  |  | -0.000 |  | 0.000 |  | 1.000 |  | 1.000 |  | 1.000 |  | -0.939 |  | 0.348 |  |
| QSM-WM |  |  |  | -0.042 |  | 0.209 |  | 0.959 |  | 0.623 |  | 1.425 |  | -0.201 |  | 0.840 |  |
| QSM-GM |  |  |  | -0.209 |  | 0.300 |  | 0.811 |  | 0.431 |  | 1.413 |  | -0.698 |  | 0.485 |  |
| **Notes.** NB=Negative Binomial; LL=lower limit; UL=upper limit; ECAS=Edinburgh Cognitive and Behavioural ALS Screen; DD=disease duration; ALSFRS-R=ALS Functional Rating Scale – Revised; ICV=intracranial volume; WM=white matter; LH=left hemisphere; GM=gray matter; RH=right hemisphere. | | | | | | | | | | | | | | | | | |

| **Supplementary Table 7.** Results of the LR model addressing bilateral volumetric and QSM measures on an above- *vs.* below-cutoff performance on the ECAS-EF. | | | | | | | | | | | | | | | | | | |
| --- | --- | --- | --- | --- | --- | --- | --- | --- | --- | --- | --- | --- | --- | --- | --- | --- | --- | --- |
|  | | | | | | | | | | **95% CI** | | | |  | | | |  |
| **Predictor** | | **Slope** | | ***b*** | | ***SE*** | | **LL** | | **UL** | | ***t*** | | ***p*** | | **Predictor** | |  |
| Sex |  | F *vs.* M |  | 5.625 |  | 2.585 |  | 277 |  | 5.303 |  | 302887 |  | 2.176 |  | 0.030 |  |  |
| C9orf72 |  | Yes *vs.* No |  | 12.865 |  | 2963.181 |  | 386601 |  | 0.000 |  | - |  | 0.004 |  | 0.997 |  |  |
| DD (months) |  |  |  | -0.010 |  | 0.019 |  | 0.990 |  | 0.950 |  | 1.047 |  | -0.508 |  | 0.611 |  |  |
| ALSFRS-R |  |  |  | 0.257 |  | 0.185 |  | 1.294 |  | 0.981 |  | 2.116 |  | 1.391 |  | 0.164 |  |  |
| Total ICV |  |  |  | 0.000 |  | 0.000 |  | 1.000 |  | 1.000 |  | 1.000 |  | 1.653 |  | 0.098 |  |  |
| Volume-WM-LH |  |  |  | 0.002 |  | 0.001 |  | 1.002 |  | 1.000 |  | 1.005 |  | 1.289 |  | 0.197 |  |  |
| Volume-GM-LH |  |  |  | -0.000 |  | 0.000 |  | 1.000 |  | 0.999 |  | 1.001 |  | -0.391 |  | 0.696 |  |  |
| Volume-WM-RH |  |  |  | -0.001 |  | 0.001 |  | 0.999 |  | 0.997 |  | 1.000 |  | -1.203 |  | 0.229 |  |  |
| Volume-GM-RH |  |  |  | -0.000 |  | 0.000 |  | 1.000 |  | 0.999 |  | 1.001 |  | -0.127 |  | 0.899 |  |  |
| QSM-WM |  |  |  | 0.399 |  | 0.865 |  | 1.491 |  | 0.280 |  | 10.403 |  | 0.462 |  | 0.644 |  |  |
| QSM-GM |  |  |  | -4.358 |  | 2.055 |  | 0.013 |  | 0.000 |  | 0.193 |  | -2.120 |  | 0.034 |  |  |
| \| **Notes.** LR=logistic regression; LL=lower limit; UL=upper limit; ECAS=Edinburgh Cognitive and Behavioural ALS Screen; EF=Executive Functioning; F=female; M=male; DD=disease duration; ALSFRS-R=ALS Functional Rating Scale – Revised; ICV=intracranial volume; WM=white matter; LH=left hemisphere; GM=gray matter; RH=right hemisphere. Effects refer to the prediction of an above-cutoff performance *vs.* a below-cutoff one. \| \| --- \| | | | | | | | | | | | | | | | | | |  |
|  | | | | | | | | | | | | | | | | | |  |

| **Supplementary Table 8.** Results of the linear model addressing bilateral volumetric and QSM measures on the *Backward digit span* task of the ECAS-EF. | | | | | | | | | | | | | | | |
| --- | --- | --- | --- | --- | --- | --- | --- | --- | --- | --- | --- | --- | --- | --- | --- |
|  | | | | | | | | **95% CI** | | | |  | | | |
| **Predictor** | | **Slope** | | ***b*** | | ***SE*** | | **LL** | | **UL** | | ***t*** | | ***p*** | |
| Sex |  | Female *vs.* Male |  | 0.023 |  | 0.492 |  | -0.942 |  | 0.988 |  | 0.048 |  | 0.962 |  |
| C9orf72 |  | Yes *vs.* No |  | 0.877 |  | 0.877 |  | -0.842 |  | 2.595 |  | 0.999 |  | 0.323 |  |
| Age (years) |  |  |  | -0.011 |  | 0.024 |  | -0.058 |  | 0.036 |  | -0.467 |  | 0.643 |  |
| Education (years) |  |  |  | 0.154 |  | 0.052 |  | 0.052 |  | 0.257 |  | 2.942 |  | 0.005 |  |
| DD (months) |  |  |  | 0.017 |  | 0.006 |  | 0.005 |  | 0.028 |  | 2.892 |  | 0.006 |  |
| ALSFRS-R |  |  |  | 0.036 |  | 0.036 |  | -0.034 |  | 0.106 |  | 1.003 |  | 0.321 |  |
| Total ICV |  |  |  | 0.000 |  | 0.000 |  | 0.000 |  | 0.000 |  | 3.388 |  | 0.002 |  |
| Volume-WM-LH |  |  |  | -0.001 |  | 0.000 |  | -0.001 |  | -0.000 |  | -2.120 |  | 0.040 |  |
| Volume-GM-LH |  |  |  | 0.000 |  | 0.000 |  | -0.000 |  | 0.000 |  | 0.852 |  | 0.399 |  |
| Volume-WM-RH |  |  |  | 0.000 |  | 0.000 |  | -0.000 |  | 0.000 |  | 0.399 |  | 0.692 |  |
| Volume-GM-RH |  |  |  | -0.000 |  | 0.000 |  | -0.000 |  | 0.000 |  | -0.911 |  | 0.367 |  |
| QSM-WM |  |  |  | -0.078 |  | 0.201 |  | -0.471 |  | 0.316 |  | -0.387 |  | 0.701 |  |
| QSM-GM |  |  |  | -0.740 |  | 0.275 |  | -1.279 |  | -0.201 |  | -2.692 |  | 0.010 |  |
| **Notes.** LL=lower limit; UL=upper limit; ECAS=Edinburgh Cognitive and Behavioural ALS Screen; EF=Executive Functioning; DD=disease duration; ALSFRS-R=ALS Functional Rating Scale – Revised; ICV=intracranial volume; WM=white matter; LH=left hemisphere; GM=gray matter; RH=right hemisphere. | | | | | | | | | | | | | | | |

| **Supplementary Table 9.** Results of the linear model addressing bilateral volumetric and QSM measures on the *Sentence completion* task of the ECAS-EF. | | | | | | | | | | | | | | | |
| --- | --- | --- | --- | --- | --- | --- | --- | --- | --- | --- | --- | --- | --- | --- | --- |
|  | | | | | | | | **95% CI** | | | |  | | | |
| **Predictor** | | **Slope** | | ***b*** | | ***SE*** | | **LL** | | **UL** | | ***t*** | | ***p*** | |
| Sex |  | Female *vs.* Male |  | 0.775 |  | 0.716 |  | -0.628 |  | 2.178 |  | 1.083 |  | 0.285 |  |
| C9orf72 |  | Yes *vs.* No |  | -0.330 |  | 1.275 |  | -2.829 |  | 2.169 |  | -0.259 |  | 0.797 |  |
| Age (years) |  |  |  | -0.014 |  | 0.035 |  | -0.083 |  | 0.054 |  | -0.412 |  | 0.682 |  |
| Education (years) |  |  |  | 0.195 |  | 0.076 |  | 0.046 |  | 0.345 |  | 2.558 |  | 0.014 |  |
| DD (months) |  |  |  | 0.004 |  | 0.009 |  | -0.013 |  | 0.021 |  | 0.490 |  | 0.626 |  |
| ALSFRS-R |  |  |  | -0.006 |  | 0.052 |  | -0.108 |  | 0.096 |  | -0.116 |  | 0.909 |  |
| Total ICV |  |  |  | -0.000 |  | 0.000 |  | -0.000 |  | 0.000 |  | -0.049 |  | 0.961 |  |
| Volume-WM-LH |  |  |  | 0.000 |  | 0.000 |  | -0.001 |  | 0.001 |  | 0.276 |  | 0.784 |  |
| Volume-GM-LH |  |  |  | 0.000 |  | 0.000 |  | -0.000 |  | 0.000 |  | 0.016 |  | 0.988 |  |
| Volume-WM-RH |  |  |  | 0.000 |  | 0.000 |  | -0.000 |  | 0.001 |  | 0.532 |  | 0.598 |  |
| Volume-GM-RH |  |  |  | 0.000 |  | 0.000 |  | -0.000 |  | 0.000 |  | 0.125 |  | 0.901 |  |
| QSM-WM |  |  |  | 0.360 |  | 0.292 |  | -0.213 |  | 0.932 |  | 1.231 |  | 0.225 |  |
| QSM-GM |  |  |  | -0.354 |  | 0.400 |  | -1.137 |  | 0.429 |  | -0.886 |  | 0.381 |  |
| **Notes.** LL=lower limit; UL=upper limit; ECAS=Edinburgh Cognitive and Behavioural ALS Screen; EF=Executive Functioning; DD=disease duration; ALSFRS-R=ALS Functional Rating Scale – Revised; ICV=intracranial volume; WM=white matter; LH=left hemisphere; GM=gray matter; RH=right hemisphere. | | | | | | | | | | | | | | | |

| **Supplementary Table 10.** Results of the NB model addressing bilateral volumetric and QSM measures on the number of errors on the *Alternation* task of the ECAS-EF. | | | | | | | | | | | | | | | | | |
| --- | --- | --- | --- | --- | --- | --- | --- | --- | --- | --- | --- | --- | --- | --- | --- | --- | --- |
|  | | | | | | | | | | **95% CI** | | | |  | | | |
| **Predictor** | | **Slope** | | ***b*** | | ***SE*** | | **OR** | | **LL** | | **UL** | | ***z*** | | ***p*** | |
| Sex |  | Female *vs.* Male |  | -0.901 |  | 0.440 |  | 0.406 |  | 0.162 |  | 0.969 |  | -2.046 |  | 0.041 |  |
| C9orf72 |  | Yes *vs.* No |  | -0.808 |  | 1.178 |  | 0.446 |  | 0.020 |  | 3.730 |  | -0.686 |  | 0.493 |  |
| Age (years) |  |  |  | 0.015 |  | 0.022 |  | 1.015 |  | 0.969 |  | 1.063 |  | 0.649 |  | 0.516 |  |
| Education (years) |  |  |  | -0.175 |  | 0.055 |  | 0.839 |  | 0.748 |  | 0.932 |  | -3.180 |  | 0.001 |  |
| DD (months) |  |  |  | -0.004 |  | 0.009 |  | 0.996 |  | 0.974 |  | 1.011 |  | -0.386 |  | 0.699 |  |
| ALSFRS-R |  |  |  | 0.010 |  | 0.031 |  | 1.010 |  | 0.950 |  | 1.075 |  | 0.321 |  | 0.748 |  |
| Total ICV |  |  |  | -0.000 |  | 0.000 |  | 1.000 |  | 1.000 |  | 1.000 |  | -2.102 |  | 0.036 |  |
| Volume-WM-RH |  |  |  | 0.000 |  | 0.000 |  | 1.000 |  | 1.000 |  | 1.000 |  | 1.283 |  | 0.199 |  |
| Volume-GM-RH |  |  |  | -0.000 |  | 0.000 |  | 1.000 |  | 1.000 |  | 1.000 |  | -0.967 |  | 0.333 |  |
| Volume-WM-LH |  |  |  | -0.000 |  | 0.000 |  | 1.000 |  | 0.999 |  | 1.000 |  | -0.603 |  | 0.547 |  |
| Volume-GM-LH |  |  |  | 0.000 |  | 0.000 |  | 1.000 |  | 1.000 |  | 1.000 |  | 0.963 |  | 0.336 |  |
| QSM-WM |  |  |  | -0.049 |  | 0.190 |  | 0.953 |  | 0.644 |  | 1.414 |  | -0.255 |  | 0.799 |  |
| QSM-GM |  |  |  | 0.217 |  | 0.244 |  | 1.242 |  | 0.797 |  | 1.947 |  | 0.889 |  | 0.374 |  |
| **Notes.** NB=Negative Binomial; LL=lower limit; UL=upper limit; ECAS=Edinburgh Cognitive and Behavioural ALS Screen; EF=Executive Functioning; DD=disease duration; ALSFRS-R=ALS Functional Rating Scale – Revised; ICV=intracranial volume; WM=white matter; LH=left hemisphere; GM=gray matter; RH=right hemisphere. | | | | | | | | | | | | | | | | | |

| **Supplementary Table 11.** Results of the NB model addressing bilateral volumetric and QSM measures on the number of errors on the *Social cognition* task of the ECAS-EF. | | | | | | | | | | | | | | | | | |
| --- | --- | --- | --- | --- | --- | --- | --- | --- | --- | --- | --- | --- | --- | --- | --- | --- | --- |
|  | | | | | | | | | | **95% CI** | | | |  | | | |
| **Predictor** | | **Slope** | | ***b*** | | ***SE*** | | **OR** | | **LL** | | **UL** | | ***z*** | | ***p*** | |
| Sex |  | Female *vs.* Male |  | -0.522 |  | 0.562 |  | 0.594 |  | 0.198 |  | 1.776 |  | -0.928 |  | 0.354 |  |
| C9orf72 |  | Yes *vs.* No |  | 0.020 |  | 1.307 |  | 1.021 |  | 0.041 |  | 11.253 |  | 0.016 |  | 0.988 |  |
| Age (years) |  |  |  | 0.097 |  | 0.030 |  | 1.102 |  | 1.041 |  | 1.173 |  | 3.201 |  | 0.001 |  |
| Education (years) |  |  |  | 0.029 |  | 0.060 |  | 1.029 |  | 0.905 |  | 1.171 |  | 0.478 |  | 0.633 |  |
| DD (months) |  |  |  | 0.004 |  | 0.006 |  | 1.004 |  | 0.987 |  | 1.022 |  | 0.682 |  | 0.496 |  |
| ALSFRS-R |  |  |  | -0.052 |  | 0.037 |  | 0.950 |  | 0.872 |  | 1.033 |  | -1.379 |  | 0.168 |  |
| Total ICV |  |  |  | -0.000 |  | 0.000 |  | 1.000 |  | 1.000 |  | 1.000 |  | -0.638 |  | 0.524 |  |
| Volume-WM-LH |  |  |  | -0.000 |  | 0.000 |  | 1.000 |  | 0.999 |  | 1.000 |  | -1.246 |  | 0.213 |  |
| Volume-GM-LH |  |  |  | 0.000 |  | 0.000 |  | 1.000 |  | 1.000 |  | 1.000 |  | 0.087 |  | 0.930 |  |
| Volume-WM-RH |  |  |  | 0.000 |  | 0.000 |  | 1.000 |  | 1.000 |  | 1.001 |  | 0.517 |  | 0.605 |  |
| Volume-GM-RH |  |  |  | 0.000 |  | 0.000 |  | 1.000 |  | 1.000 |  | 1.000 |  | 0.534 |  | 0.593 |  |
| QSM-WM |  |  |  | 0.183 |  | 0.229 |  | 1.200 |  | 0.748 |  | 1.980 |  | 0.797 |  | 0.426 |  |
| QSM-GM |  |  |  | 0.895 |  | 0.310 |  | 2.448 |  | 1.356 |  | 4.750 |  | 2.888 |  | 0.004 |  |
| **Notes.** NB=Negative Binomial; LL=lower limit; UL=upper limit; ECAS=Edinburgh Cognitive and Behavioural ALS Screen; EF=Executive Functioning; DD=disease duration; ALSFRS-R=ALS Functional Rating Scale – Revised; ICV=intracranial volume; WM=white matter; LH=left hemisphere; GM=gray matter; RH=right hemisphere. | | | | | | | | | | | | | | | | | |

|  |
| --- |
